# Colorectal Cancer: Prevention and Management of Metastatic Disease

**DOI:** 10.1155/2014/782890

**Published:** 2014-03-24

**Authors:** Paul H. Sugarbaker

**Affiliations:** Washington Cancer Institute, 106 Irving Street, NW, Suite 3900, Washington, DC 20010, USA

## Abstract

This paper compared the similarities and differences of the two most common types of colorectal cancer metastases. The treatment of liver metastases by surgery combined with systemic chemotherapy was explained. The different natural history of liver metastases as compared to peritoneal metastases and the possibility for prevention of peritoneal metastases were emphasized. Perioperative cancer chemotherapy or second-look surgery must be considered as individualized treatments of selected patients who have small volume peritoneal metastases or who are known to be at risk for subsequent disease progression on peritoneal surfaces. However, the fact that peritoneal metastases, when diagnosed in the follow-up of colorectal cancer patients, can be cured with a combination of cytoreductive surgery and hyperthermic perioperative chemotherapy cannot be ignored. Careful follow-up and timely intervention in colorectal cancer patients with progressive disease are a necessary part of the management strategies recommended by the multidisciplinary team. After a critical evaluation of the data currently available, these strategies for prevention and management of colorectal metastases are presented as the author's recommendations for a high standard of care. As more information becomes available, modifications may be necessary.

## 1. Introduction

The most effective strategy to combat disease is prevention. Prevention involves the identification of high risk groups and application of effective management strategies in the early stage of the disease. Because there are different manifestations of colorectal cancer metastatic disease, the strategies employed in the treatment of the primary malignancy must be individualized. The surgery that is performed must provide a complete CLEARANCE of the primary cancer and its lymph node groups at risk for metastatic disease. The resection must be accomplished with perfect CONTAINMENT of the process. The surgeon must be aware of the fact that patients may enter the operating room with a contained colorectal malignancy and leave, as a result of surgical trauma, with disseminated disease. This situation results from trauma to the cancer specimen so that malignant cells are lost from the specimen into the resection site or free peritoneal cavity. Cancer cells lost into the resection site result in local-regional recurrence; cancer cells lost into the free peritoneal cavity result in peritoneal metastases. This dissemination of the malignant process can occur with open colorectal surgery or with laparoscopic resection.

This paper is not a commentary on the 70% of patients who have an early and uncomplicated colorectal cancer resection with a favorable prognosis. It concerns the approximately 30% of patients who have advanced disease that must be identified at the time of the disease presentation. It also involves the management of the approximately 50% of patients who, months or years after resection, manifest treatment failure with the diagnosis of progressive disease. Emphasis is on prevention of the metastatic process or an attempt to contain the metastatic process diagnosed in follow-up of the colorectal cancer patient in a timely manner.

### 1.1. A Comparison and Contrast of Liver Metastases and Peritoneal Metastases

The two most common sites for surgical treatment failure with colorectal malignancy are liver metastases and peritoneal metastases. Although these sites for progression may occur in the same patient, frequently liver metastases are isolated and can be surgically resected with curative intent. Also, peritoneal metastases may occur to a limited extent and in the absence of other sites of metastatic disease; in this clinical situation treatment for cure should also be explored.  Table 1 lists the comparisons and contrasts of liver metastases and peritoneal metastases. The route of dissemination is decidedly different. Liver metastases result from cancer cells being released from the primary malignancy into the portal blood. Single cells or small clusters of cancer cells then lodge within the venous sinusoids of the liver. Over time, they become vascularized by the hepatic artery blood flow. They progress as masses within the parenchyma of the liver until they reach a size that results in necrosis of the mass. With this disruption of the capillaries within the liver metastasis, cells can be released into the systemic circulation, especially into the lung pulmonary vasculature to result in lung metastases and other sites of systemic disease.

The mechanism of dissemination of peritoneal metastases is by direct extension of the primary malignancy into the free peritoneal space. This can occur as a result of full-thickness invasion of the bowel wall by the primary malignancy. When this occurs, the surgeon will find peritoneal metastases in the vicinity of the primary malignancy, layered out under the right hemidiaphragm or involving the pelvic peritoneum. Despite the fact that peritoneal metastases are present, cytological study of the peritoneal fluid is often negative. In women, a frequent site of the progression of peritoneal metastases is the ovaries, especially in the premenopausal woman.

**Figure 1:**
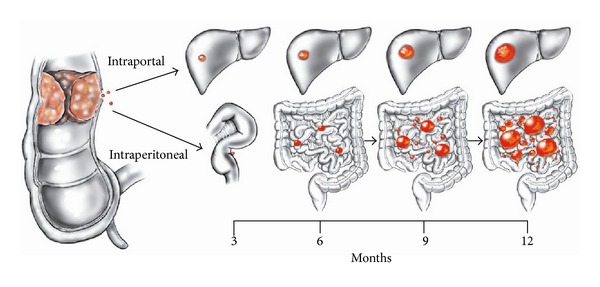
Schematic diagram that presents a theoretical model comparing the progression of one colorectal liver metastasis to one peritoneal metastasis over one year. The liver metastasis will expand within the liver parenchyma with a doubling time of approximately three months. The peritoneal metastasis will progress at approximately the same speed but will also exfoliate cancer cells into the free peritoneal space. Many cancer nodules of many different sizes will occur, widely distributed throughout the abdomen and pelvis within one year (reprinted with permission from Sugarbaker PH. Cytoreductive surgery plus hyperthermic perioperative chemotherapy for selected patients with peritoneal metastases from colorectal cancer: a new standard of care or an experimental approach? Gastroenterol Res Pract Volume 2012; 2012: Article ID 309417, 9 pages).

The mode of progression of liver metastases is by direct expansion of the metastatic site within the liver parenchyma. The doubling time has been estimated at approximately 3 months. Only as the liver metastasis becomes quite large, generally over 10 cm, do satellite liver metastases form as a result of cancer emboli within liver lymphatics [1]. Metastases to peritoneal surfaces also have a doubling time estimated at approximately 3 months [1]. The peritoneal nodules expand in a predictable manner over time. However, peritoneal metastases have an alternative and more aggressive mechanism of abdominal and pelvic progression. The nature of the epithelial cell that gives rise to the adenocarcinoma is to exfoliate from the epithelial surface. Also, peritoneal metastases, even small nodules of cancer, will exfoliate free cancer cells into the peritoneal space. This exfoliation process may cause a more rapid disease progression with all quadrants of the abdomen being brought into the metastatic process within a few months.  Figure 1 illustrates this comparison of the progression of a single liver metastasis and a single peritoneal metastasis over a period of 1 year.

The metastatic efficiency of liver metastases is extremely low. The portal venous blood may be contaminated by millions of cancer cells and yet only a few implants grow within the liver parenchyma. In marked contrast, the metastatic efficiency of peritoneal metastases is extremely high, exponentially different from the implantation of cancer cells within the portal blood [2]. In some situations in which the peritoneum has been irritated to create a “sticky site,” the implantation may be as high as 1 : 1. It has been shown that the trauma produced by an operative intervention may greatly increase the efficiency of cancer cell implantation within the peritoneal space [3].

With a primary colon cancer, the surgeon will encounter liver metastases in approximately 20% of patients as compared to 10% of patients with peritoneal metastases. In patients with recurrent disease, one estimates that 50% will have liver metastases. However, the incidence of peritoneal metastases at the time of recurrence is even higher than the incidence of liver metastases. For lack of an alternative explanation, cancer cells disseminated as part of the resection of the primary malignancy account for this steep increase in the incidence of peritoneal metastases observed in the follow-up of recurrent malignancy.

There is a definite difference in the response of liver metastases to modern systemic chemotherapy. Approximately 60% of liver metastases will respond and about half that number will completely disappear [4]. In contrast, only about 30% of patients with peritoneal metastases will respond to modern systemic chemotherapy with 15% or less showing a complete response [5].

If reoperative surgery with resection of metastatic disease is to profit a patient with recurrent colorectal cancer, an R-0 resection is required of both the liver metastases and the peritoneal metastases. However, with current modern systemic chemotherapy the number of liver metastases has less impact on prognosis. The long-term survival with single liver metastases or multiple liver metastases shows benefit as long as there is an R-0 resection [4].

Benefit from resection of peritoneal metastases is greatly dependent upon the extent of disease even though there is complete cytoreduction with no visible evidence of disease at the completion of the cytoreductive surgery. The peritoneal cancer index (PCI) is most often used to estimate the extent of disease [6].

At this point in time no effective prevention strategies exist for liver metastases. Portal vein infusion shows promise as investigated by Laffer and colleagues but was never placed into general practice [7]. However, systemic chemotherapy is currently used in an attempt to decrease the incidence of liver metastases. Data to support this assumption does not exist. In contrast, strategies to prevent peritoneal metastases in groups of patients at high risk for local-regional and peritoneal implantation of cancer cells at the time of a colorectal cancer resection have been described. Also, special resection technologies in order to minimize the spillage of cancer cells that result from colon or rectal resection have been extensively documented [8–11].

This comparison and contrast of liver metastases and peritoneal metastases strongly suggests that individualized management strategies for these two different manifestations of colorectal cancer progression should be exercised. Systemic chemotherapy alone to treat all manifestations of progressive disease is below the standard of practice. A strong difference between peritoneal metastases and liver metastases comes from the possible prevention of peritoneal metastases but the lack of strategies to prevent the occurrence of liver metastases.

### 1.2. Management of Hepatic Metastases from Colorectal Cancer

As a result of the pioneering efforts of Wilson and Adson [12], Foster and Berman [13], and Hughes and colleagues [14], the benefits to be expected with resection of liver metastases from colorectal cancer have been clearly established and liver resection for metastatic disease is a standard of practice. This standard of care has come about despite the fact that there is no verification of this practice from phase III and randomized controlled studies [15]. Overall, multiple single institution and multi-institution reports document the survival following an R-0 liver resection between 30% and 50% at 5 years. There may be some improvement in this statistic as a result of repeat hepatic resections that have been shown to be successful [16, 17]. Also, in some patients with liver metastases that are unresectable because of the large extent of disease, systemic chemotherapy can be used to downsize the liver metastases so that an R-0 resection is possible. The survival of this group of patients receiving neoadjuvant chemotherapy who go on to have an R-0 resection is nearly identical to patients having hepatic resection as the initial treatment [4].

Patient-related factors associated with a reduced survival include increased serum carcinoembryonic antigen (CEA), positive lymph node status of the primary tumor, lymph nodes present in the regional portal lymphatic system, and a disease-free interval from primary resection to hepatic resection of less than 1 year. Clinical features of the liver metastases that carry a poor prognosis include the following: More than 6 hepatic lesions, increasing size of the largest lesions, bilobar distribution, and percentage of hepatic parenchyma replaced by cancer. Finally, technical factors such as a positive or close margin of resection will carry a reduced prognosis [18].

### 1.3. Prevention of Peritoneal Metastases

Recent improvements in the surgical technology of colorectal cancer resection have decreased the incidence of treatment failures, both at the resection site or at a distance from the primary. The benefits of total mesorectal excision have been established and the survival benefit published [8, 9]. This survival advantage has been a result of the absence of tumor contamination within the confines of the pelvis because of a meticulous dissection which maintains a layer of tissue between the primary malignancy and the margins of resection [9]. Also, the benefits of colon cancer resection using wide excision, generous lymphadenectomy, and an intact mesocolic resection have been demonstrated [11]. These improvements in surgical technology and therefore in survival are the result of decreased tumor cell contamination resulting from the surgical event itself. A complete absence of tumor cell contamination with primary colorectal cancer surgery has become an absolute requirement of treatment. Any dependence upon systemic chemotherapy to manage resection site disease or peritoneal metastases must be abandoned.

**Figure 2:**
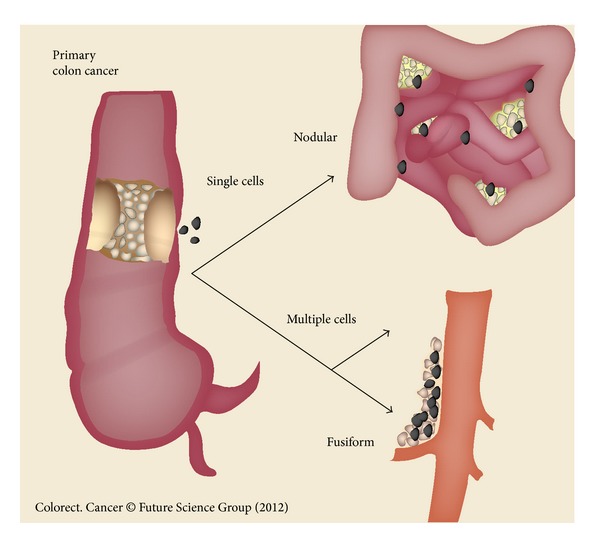
Anatomic sites of right colon cancer progression by the dissemination of cancer cells or minute nodules. The mechanism for right colon cancer implantation and progression at the cancer resection site along the superior mesenteric vessels or on peritoneal surfaces is similar (reprinted with permission from Sugarbaker PH, Sammartino P, and Tentes AA. Proactive management of peritoneal metastases from colorectal cancer: the next logical step toward optimal locoregional control. Colorect Ca 2012; 1 : 115–123).

It is important to establish that the mechanism of resection site recurrence and peritoneal metastases is the same. Cancer cells are disseminated either prior to or at the time of the cancer resection. The cancer cells at high density will layer out within the bed of the resection site. Because the surgery has disrupted the peritoneum and created a “sticky surface,” a high metastatic efficiency is expected. Single cells disseminated at a distance from the anatomic site of primary cancer resection will progress as peritoneal metastases.  Figure 2 illustrates anatomic sites of right colon cancer progression by the dissemination of cancer cells or minute cancer nodules at the time of surgery. The mechanism whereby cancer cells recur within the abdominal incision, within the resection site along the superior mesenteric vessels, or on peritoneal surfaces is similar.

There are clinical findings in approximately 30% of primary colorectal cancer patients present at the time of primary cancer resection that indicate that there is a high likelihood of cancer cell contamination. These patients need individualized treatments to prevent local-regional recurrence and peritoneal metastases. These clinical findings suggest that the primary colorectal cancer surgery, even performed in its most perfect manner with or without systemic chemotherapy, is not a sufficient management strategy. If a high risk of local-regional recurrence or peritoneal metastases is evident, specialized additional treatments need to be added to the management strategy of the primary malignancy. These patients at special risk for local-regional recurrence and peritoneal metastases are listed in Table 2. In groups 1–4 in Table 2, patients can be considered to have 50–100% incidence of local-regional recurrence and/or peritoneal metastases in the absence of special treatments. Peritoneal metastases documented at the time of primary colorectal cancer resection will show progression with follow-up in 75% of patients even if these metastases are completely removed with the primary intervention [19]. Ovarian metastases, even if resected with oophorectomy and/or hysterectomy have over 60% incidence of other sites of peritoneal dissemination in follow-up. Perforation through the primary cancer at the time of primary cancer resection and a positive margin of resection, usually a lateral margin, indicates a likelihood of local-regional or peritoneal progression in 54 and near 100% of patients, respectively.

The other clinical findings (# 5–10 listed in Table 2) have been shown to place the patient at risk for local-regional recurrence or peritoneal metastases but at a lesser incidence. Positive peritoneal cytology either before or after colorectal cancer resection, adjacent organ involvement or a cancer-induced fistula, T3 mucinous cancers, T4 cancers or a positive imprint cytology from the primary malignancy, rupture of the cancerous mass, or obstruction at the time of presentation all would have an elevated incidence of local-regional recurrence and peritoneal metastases [20].

### 1.4. Data Showing Benefit from Perioperative Chemotherapy in Patients with Primary Colorectal Cancer with Peritoneal Seeding or at High Risk for Peritoneal Seeding

Oncologists are well aware of the prominent role that local-regional recurrence and peritoneal metastases have occupied in the natural history of gastrointestinal cancer. Chemotherapy in the abdomen used as a planned part of a surgical intervention to control local-regional recurrence and peritoneal dissemination from colorectal cancer was proposed by Sugarbaker and colleagues [20–22]. They performed phase I/II studies with 5-fluorouracil and mitomycin C administered directly into the peritoneal cavities in the early postoperative period before adhesions had progressed. There was a marked pharmacokinetic advantage of perioperative intraperitoneal chemotherapy with single cancer cells on peritoneal surfaces as the targets of this treatment [23].

Experience with patients demonstrating peritoneal metastases recognized at the time of primary colon cancer resection came from Washington, DC, and was reported by Pestieau and Sugarbaker [24]. They identified five patients who had definitive treatment of peritoneal metastases from colon cancer concomitant with the resection of the primary tumor. At the time of writing this paper, the median disease-free survival of these patients had not been reached and their 5-year survival was 100%. The statistical difference between patients who had perioperative treatment of their peritoneal metastases as compared to those who had delayed management with cytoreductive surgery and early postoperative intraperitoneal chemotherapy (EPIC) was statistically significant (*P* < 0.0001) (Table 3).

Tentes has reported their experience on the use of hyperthermic perioperative chemotherapy in patients at high risk for local-regional recurrence. These were patients with locally advanced T3 or T4 colorectal cancer. Only patients with R-0 resection were randomly assigned to receive HIPEC plus systemic chemotherapy versus conventional treatments, which were surgery plus systemic adjuvant chemotherapy. The 5-year survival for the HIPEC group was 100% and 72% for the conventional group. The difference in survival showed a trend toward significance (*P* = 0.0938). During follow-up, 2 patients in the HIPEC group and 8 patients in the conventional group were recorded with recurrence (*P* = 0.002). It is important to note that no local-regional recurrence or peritoneal metastases was recorded in the HIPEC group. By contrast, the group treated in a conventional manner showed 3 patients with local-regional recurrence. These authors suggest that the perioperative chemotherapy had no effect on the development of distant metastases but exhibited an advantage in eradicating viable cancer cells that were disseminated local-regionally at the time or prior to the colorectal cancer resection [25].

Noura and colleagues reporting from the cities of Osaka and Sakai, Japan, reported on colorectal cancer patients with no clinically confirmed peritoneal metastases but a positive peritoneal lavage cytology. Thirty-one of 52 patients with positive cytology were treated by mitomycin C instillation through catheters after abdominal closure. Patients receiving perioperative chemotherapy had a significantly improved survival rate (*P* < 0.05). In a multivariate analysis, perioperative chemotherapy remained an independent prognostic factor for peritoneal recurrence-free survival [26].

Braam and colleagues from Nieuwegein, The Netherlands, reported on a total of 72 patients with synchronous peritoneal metastases from colorectal cancer. In 20 patients (27.8%) the primary tumor was resected simultaneously with HIPEC (early referral). In the other 52 patients (72.2%) the primary tumor was resected prior to the HIPEC procedure (late referral). During CRS plus HIPEC following late referral, 22 (59.5%) of the 37 anastomoses of the earlier operation were resected, revealing malignancy in 12 patients (54.5%) on histopathological examination. In twenty (27.8%) patients a permanent colostomy was constructed after HIPEC. Ten of these patients had complete bowel continuity after earlier primary resection. The relaparotomy rate was higher in patients after a resection of a previous anastomosis (36.4%) compared to 12% in the rest of the patients (*P* = 0.02). Resection of the primary tumor simultaneously with HIPEC in patients with synchronous peritoneal metastases from colorectal cancer may prevent extended bowel resections and permanent colostomy. These data support early referral of patients with PC from colorectal cancer [27].

Sammartino and colleagues from Rome studied colon cancer patients with clinical T3/T4, any N, M0 stage, and mucinous histology or signet ring histology [28]. Twenty-five patients in the experimental group underwent carcinomatosis prevention strategies including complete omentectomy, bilateral salpingo-oophorectomy, hepatic round ligament resection, and appendectomy. At the end of the colorectal cancer resection plus carcinomatosis prevention resections, hyperthermic intraperitoneal chemotherapy using intraperitoneal oxaliplatin with intravenous fluorouracil was administered. These experimental patients were compared with 50 matched controlled patients. All patients had an R-0 resection. The morbidity of the two groups of patients was the same. At 48 months, after the study ended, fewer patients in the proactive group than in the control group had recurrent disease (28% versus 42%). Peritoneal metastases and local recurrence developed significantly less often in the proactive group than in the control group (4% versus 28%, *P* < 0.03). Median survival was 59.5 months among the patients included in the proactive treatment and 52 months in the control group. The disease-free survival in the two groups was different with *P* < 0.04. The overall survival in the two groups was different with *P* < 0.03.

To date, the optimal perioperative chemotherapy treatment for prevention of local-regional recurrence and peritoneal metastases has not been determined. It is possible that the best choice is the early postoperative intraperitoneal chemotherapy. This was used by Pestieau and Sugarbaker to achieve good results [24]. Also, in the prevention of peritoneal metastases in gastric cancer, EPIC was shown by Yu et al. to be very successful in a prospective randomized controlled study [29]. From a logistical perspective, EPIC may be favored in that patients with unexpected peritoneal metastases who have not signed an informed consent for HIPEC can be treated with full consent in the early postoperative period. It is possible that a single dose of intraoperative chemotherapy (HIPEC) is not as effective as the 5-day intraperitoneal lavage used postoperatively (EPIC). However, EPIC has been shown to be associated with a higher incidence of adverse events but not a higher incidence of mortality [30].

## 2. Current Data regarding Benefits Expected with Proactive Second-Look Surgery

In patients treated for primary CRC in institutions where cytoreductive surgery and HIPEC are not available, a second strategy for proactive management of patients at high risk for progression of peritoneal metastases must be formulated. The inclusion criteria for the patients included in this clinical pathway are those listed in Table 2. Patients in groups 1–4 are those who must be recommended for a repeat surgical intervention (proactive second-look surgery) if a high likelihood of long-term survival as a result of optimal treatment is expected. Patients in the high risk groups 5–10 need to be carefully monitored; laparoscopy rather than laparotomy may be recommended for a planned second-look intervention.

In the USA a long history of efforts to use second-look surgery to improve the survival rate of colorectal cancer patients has been accumulated in the surgical literature. Griffen and colleagues first organized a planned approach of reoperative surgery in asymptomatic gastrointestinal cancer patients [31]. Minton and colleagues revisited this problem suggesting that second-look should be initiated by patients' symptoms (symptomatic second-look) or a progressive increase in serial carcinoembryonic antigen (CEA) assays obtained in follow-up [32].

The history of second-look surgery and its application in modern surgical oncology has been recently reviewed [33]. Two important changes in the second-look treatment strategy have occurred. First, patients selected for a repeat intervention in the absence of signs or symptoms of progressive disease are those patients listed in groups 1–4 of Table 2. The second important change is that this second-look would be combined with cytoreductive surgery plus peritonectomy and HIPEC as a planned part of the repeat intervention.

The evaluation of this revised strategy for the use of second-look surgery must be prospective and thorough. The primary endpoint for the study is the percentage of patients who have a positive second-look with an R-0 resection and as a result of the repeat surgical intervention enjoy long-term survival. To use Wangensteen terminology, these are patients “converted” from disease documented at the time of second-look surgery to a 5-year survival [31]. A second endpoint would be the percentage of patients who had a negative second-look. This would provide an estimate of patients who had “unnecessary surgery” as result of the elective reintervention. Of course, a third endpoint would be a comprehensive morbidity and mortality assessment of both positive and negative second-look procedures.

Elias and colleagues from Villejuif, France, published their experience with second-look surgery for colorectal cancer patients at high risk for progression [34]. This was a highly selected group of patients who had biopsy-proven peritoneal metastases, ovarian peritoneal metastases, or perforation confirmed at the time of primary colorectal cancer resection. The second-look surgery was performed within 1 year after the first surgery and after the completion of systemic adjuvant chemotherapy. The patients treated by Elias were asymptomatic with a completely negative work-up. The authors detected additional peritoneal metastases in 63% of patients who had synchronous peritoneal metastases, 75% of patients with ovarian metastases, and 33% of patients with a perforated primary tumor. Patients with macroscopic peritoneal metastases were treated with cytoreductive surgery plus HIPEC with no mortality, a low morbidity, and a 2-year disease-free survival rate exceeding 50%. Patients without macroscopic peritoneal metastases received prophylactic peritoneal metastases surgery with or without HIPEC. It is interesting to note that, in this subgroup with no macroscopic peritoneal metastases, 17% who received HIPEC showed recurrence versus 43% showed recurrence who did not receive HIPEC.

At the MedStar Washington Cancer Institute, Washington, DC, we have early results with 20 patients who have had proactive management with colon cancer. Upon reoperation in these 20 patients, 62% had a peritoneal cancer index between 1 and 10. Also, 85% had complete cytoreduction with the second-look surgery. In our patients who had only 4 cycles of chemotherapy prior to the second-look surgery, all 20 patients (100%) were found to have progressive peritoneal metastases. The long-term survival of these patients with a low peritoneal cancer index and a complete cytoreduction is 60% [35].

Delhorme et al. from Strasbourg have published data on a mandatory second-look surgery for the treatment of histologically confirmed peritoneal metastases present with the primary colon cancer resection. At the time of their proactive second-look surgery, 71% of patients were found to have persistent or progressive disease and the median peritoneal carcinomatosis index was 10. There was no postoperative mortality and there was a 7% incidence of grade III/IV complications. The 2-year overall survival and disease-free survival rates were 91% and 38%, respectively. Following proactive second-look surgery with HIPEC, peritoneal recurrence was observed in only 8% of patients versus 100% of the patients treated in a standardized fashion [36].

## 3. Update on Strategies for Proactive Surgical Management

The new concepts regarding the mechanism for local recurrence and peritoneal metastases must change the surgical technologies for the current management of primary colorectal cancer. First, extreme care is taken with primary colorectal cancer resection to prevent trauma to the cancer specimen as it is removed. The concepts of total clearance and total containment of the malignant process during the cancer resection have been shown to be imperative [8]. A total mesorectal resection and a total mesocolic resection are the surgical requirements for containment of the malignancy during intervention to remove the colon or rectal cancer [9, 11]. Also, total relevant lymphadenectomy with lymph node resections carried down to the superior mesenteric artery and vein on the right or aorta on the left has supporting data [11].

In patients with primary colorectal cancer resection, ten clinical features (Table 2) are used to identify patients who are eligible for treatment with hyperthermic perioperative. At institutions where perioperative chemotherapy is not available, proactive second-look surgery on patients shown to have visible evidence of peritoneal metastases, ovarian cyst showing adenocarcinoma, perforation, or positive margins of resection should be recommended after treatment with systemic chemotherapy. These proactive treatment strategies are derived from the abundant evidence showing that the results of treatment of peritoneal metastases are in large part dependent upon the extent of disease as measured by the peritoneal cancer index at the time of definitive cytoreductive surgery with hyperthermic perioperative chemotherapy.

There are numerous technologies that must be considered for management of local-regional recurrence and peritoneal metastases. Recent experience with a large volume of irrigation has gained new interest. This technology uses 10 liters of saline one liter at a time. The concept of extensive intraoperative peritoneal lavage (EIPL) has been found to be used with benefit in patients with gastric cancer and positive peritoneal cytology. However, EIPL was most effective when combined with HIPEC [37].

There is no doubt that the surgical strategy for proactive management demands peritonectomy procedures and visceral resections to achieve no visible evidence of disease. However, it is unclear at this point in time what the optimal perioperative treatment strategy should be. Perhaps the most widely used in Europe is high dose intraperitoneal oxaliplatin with systemic 5-fluorouracil for a 30-minute HIPEC treatment. The Dutch group has used high dose mitomycin C at 35 mg/m^2^ given in three doses (1*⁄*2, 1*⁄*4, 1*⁄*4, at 30-minute intervals) over a 90-minute intraperitoneal lavage with 42°C heat. A third regimen combines a lower dose of hyperthermic mitomycin C (10–15 mg/m^2^) for a 90-minute lavage with EPIC 5-fluorouracil for four postoperative days [38]. To date, clinical trials to determine the most effective regimen with an acceptable morbidity have not been initiated.

## 4. Morbidity/Mortality for the Proactive Approach

Of course, a new initiative for the comprehensive management of peritoneal metastases should not be implemented without strong evidence that it does not add to the complications that occur in this group of patients. In the 80 randomized patients presented by Tentes, there was one in-hospital mortality in the HIPEC group and three in the conventionally treated group. There was a 32% morbidity in the HIPEC group and a 22% morbidity in the conventionally treated group. The incidence of complications was statistically and significantly higher in the HIPEC group with a *P* value less than 0.05 [25]. In the manuscript presented by Sammartino and colleagues, there was a 4% combined grade III and IV toxicity. In the control group there was an 8% incidence of grade III and IV adverse events. There were no deaths in either group [28]. In a recent review of morbidity/mortality in colorectal and appendiceal patients who have had extensive cytoreduction combined with perioperative chemotherapy, Sugarbaker and colleagues showed a 0.6% mortality and a 12% grade IV morbidity [39]. These data taken together suggests that once the learning curve has been ascended in patients who have cytoreductive surgery combined with perioperative chemotherapy, the morbidity and mortality compares favorably and is perhaps even lower than in patients who undergo advanced surgery for gastrointestinal malignancy.

## 5. Management of Peritoneal Metastases Diagnosed in Follow-Up

Survival benefits for peritoneal metastases from colon and rectal cancer using cytoreductive surgery and perioperative chemotherapy began to appear in publications in the 1990s. Although a small percentage of these patients had synchronous peritoneal metastases (less than 5%), a great majority had peritoneal metastases diagnosed in follow-up. In 1995, Sugarbaker and Jablonski showed a 3-year survival of 35% in patients with peritoneal metastases from colon cancer treated with cytoreductive surgery plus intraperitoneal mitomycin C and fluorouracil [40]. In 2003, Verwaal and colleagues from Amsterdam published a 3-year projected survival of 38% in 54 patients treated by cytoreductive surgery and hyperthermic intraperitoneal mitomycin C with adjuvant systemic 5-fluorouracil [41]. Shen and colleagues accumulated patients between 1991 and 2002 [42]. Seventy-seven patients with nonappendiceal colorectal cancer underwent the combined treatment. These investigators concluded that one-third of patients with complete resection have long-term survival and that systemic chemotherapy did not contribute to the control of peritoneal metastases. These studies performed in the absence of modern colorectal cancer chemotherapy (oxaliplatin and irinotecan) document the efficacy of cytoreductive surgery and perioperative chemotherapy to rescue approximately one-third of patients with peritoneal metastases.

Since that time, multiple publications confirming the efficacy of the combination of cytoreductive surgery and perioperative chemotherapy to benefit patients with established colorectal peritoneal metastases have been published. Glehen and colleagues, in a multi-institutional retrospective study of 506 patients from 28 institutions, reported an overall median survival of 19.2 months in patients with peritoneal metastases from colorectal cancer treated with the combined approach [30]. Patients in whom the cytoreductive surgery was complete had a median survival of 32.4 months compared with 8.4 months in patients in whom cytoreduction was not completed (*P* < 0.001). The morbidity was 22.9% and the mortality was 4%. These investigators concluded that the therapeutic approach of combining cytoreductive surgery with perioperative intraperitoneal chemotherapy achieved long-term survival in a selected group of patients with peritoneal metastases of colorectal origin with acceptable morbidity and mortality. The complete cytoreduction was the most important prognostic indicator.

Elias and colleagues reported on colorectal peritoneal metastases in a retrospective analysis of 523 patients from 23 French-speaking centers [43]. The overall median survival was 30.1 months and the 5-year overall survival was 27%. Eighty-four percent of the patients had a complete cytoreduction, with a median survival of 33 months. These investigators concluded that cytoreductive surgery and perioperative chemotherapy are now considered the gold standard in the French guidelines for management of peritoneal metastases. Similarly, Verwaal reported a long-term Dutch multicenter data analysis [44]. The survival of 562 patients at 10 years was 37%.

At the top of the list regarding evidence-based medicine for this treatment strategy is the phase 3 study reported by Verwaal and colleagues in 2003 [45]. The Dutch trial compared 105 patients with colorectal peritoneal metastases who were randomly assigned to receive either standard treatment with systemic 5-fluorouracil and leucovorin compared with an aggressive cytoreductive surgery with perioperative chemotherapy using hyperthermic mitomycin C. The patients in the experimental therapy arm also had systemic 5-fluorouracil chemotherapy. After a median follow-up of 21.6 months, the median survival was 12.6 months with systemic chemotherapy and 22.3 months with cytoreduction and perioperative chemotherapy (*P* = 0.032). These investigators reported that a complete cytoreduction and a limited extent of disease were important determinants of benefit. The durability of the benefit of cytoreductive surgery and perioperative chemotherapy was confirmed in a follow-up article in 2008 [46]. Currently, this treatment strategy is the standard of care in Holland and there are five regional centers of excellence open for peritoneal metastases patients.

Recently, these benefits have been called into question by Sugarbaker [47]. He has questioned the relevance of cytoreductive surgery and perioperative chemotherapy now that oxaliplatin, irinotecan, and molecular agents are available. He contends that the benefits of systemic chemotherapy alone are so great that cytoreduction plus perioperative chemotherapy is no longer indicated. However, current data confirm that for a limited extent of peritoneal metastases, a multidisciplinary approach using the best surgical and best chemotherapy treatments is preferable. Franko and colleagues presented data to show that these two options work best when used together [48]. They showed that the median survival was longer in patients treated by modern systemic chemotherapy when cytoreductive surgery and hyperthermic intraperitoneal chemotherapy were added to the clinical pathway. Elias and colleagues produced similar data in a retrospective study with matched colorectal peritoneal metastases patients either treated with cytoreductive surgery and HIPEC or by modern systemic chemotherapy. There were 48 patients in each group. Two-year and five-year overall survival rates were 81% and 51% for the HIPEC group, respectively, and 65% and 13% for the standard group, respectively. Median survival was 23.9 months in the standard group versus 62.7 months in the HIPEC group (*P* < 0.05, log-rank test) [49]. Currently, standard of care, until more data become available, indicates that patients with peritoneal metastases from colorectal cancer have the right to be informed of a possible curative treatment option. It is the oncologist's obligation to provide the relevant information in a timely fashion [47].

## 6. Future Directions

The future demands improved intracavitary treatment modalities that can preserve the surgical complete response. These perioperative treatments must more adequately treat small volumes of cancer cells or small established cancer nodules on peritoneal surfaces. Monoclonal antibodies such as catumaxomab may be indicated [50]. Molecular agents used with a surgical procedure to prevent angiogenesis may present an opportunity for benefit. Also, combinations of current available chemotherapy used intraoperatively, early postoperatively, and as adjuvant bidirectional chemotherapy (intravenous and intraperitoneal) need to be tested regarding their efficacy and safety in patients identified to be at high risk for progression of local-regional recurrence or peritoneal metastases [51].

## Figures and Tables

**Table 1:** Comparison and contrast of liver metastases and peritoneal metastases from colorectal cancer.

	Liver Metastases	Peritoneal Metastases
Mechanism of dissemination	Portal vein	Peritoneal space
Mode of progression	Expansion of a parenchymal mass	Exfoliation
Metastatic efficiency	Low	High
Incidence with primary resection	20%	10%
Incidence with diagnosis of recurrence	50%*	60%*
Response to modern systemic chemotherapy	60%	30%
Benefit from reoperative surgery requires an R-0 resection	Yes	Yes
Preventive strategies in existence	No	Yes

*The high incidence of peritoneal metastases seen with recurrent cancer may be in large part preventable. No known treatment to prevent liver metastases is currently available.

**Table 2:** Patients with primary colorectal cancer identified to be at high risk for local-regional recurrence and/or peritoneal metastases. Groups 1–10 are candidates for prophylactic HIPEC or EPIC as part of the primary colorectal cancer resection. Groups 1–4 are candidates for proactive second-look surgery.

(1) Visible evidence of peritoneal metastases	
(2) Ovarian cysts showing adenocarcinoma suggested to be of gastrointestinal origin	
(3) Perforated cancer	
(4) Positive margins of excision	
(5) Positive cytology either before or after cancer resection	
(6) Adjacent organ involvement of cancer-induced fistula	
(7) T3 mucinous cancer	
(8) T4 cancer or positive “imprint cytology” of the primary cancer	
(9) Cancer mass ruptured with the excision	
(10) Obstructed cancer	

**Table:** (a)

Proactive CRS and HIPEC with primary colorectal cancer					
Authors	Institution	Year	Number of patients	Treatment	Survival statistic
Pestieau and Sugarbaker [24]	Washington, DC	2000	5	EPIC	*P* < 0.0001
Tentes et al. [25]	Didimotichon, Greece	2013	41	HIPEC	*P* < 0.03
Noura et al. [26]	Osaka and Sakai, Japan	2011	31	IP MMC	*P* < 0.005
Braam et al. [27]	Nieuwegein, The Netherlands	2013	20	HIPEC	NA
Sammartino et al. [28]	Rome, Italy	2013	25	HIPEC	*P* < 0.03

**Table:** (b)

CRS and HIPEC as proactive second-look					
Authors	Institution	Year	Number of patients	Treatment	Disease-free survival
Elias et al. [34]	Villejuif, France	2008	29	HIPEC	59% at 29 months
Sugarbaker et al.	Washington, DC	2013	20	HIPEC	85% at 2 years
Delhorme et al. [36]	Strasbourg, France	2013	14	HIPEC	38% at 2 years

HIPEC: hyperthermic perioperative chemotherapy; EPIC: early postoperative intraperitoneal chemotherapy; IP MMC: intraperitoneal mitomycin C; NA: not available.
